# Not the Function of Eating, but Spontaneous Activity and Energy Expenditure, Reflected in “Restlessness” and a “Drive for Activity” Appear to Be Dysregulated in Anorexia Nervosa: Treatment Implications

**DOI:** 10.3389/fpsyg.2018.02303

**Published:** 2018-11-23

**Authors:** Regina C. Casper

**Affiliations:** Department of Psychiatry, Stanford University School of Medicine, Stanford, CA, United States

**Keywords:** anorexia nervosa, restlessness, drive for activity, energy expenditure, activity levels, restless activation, fear of weight gain

## Abstract

Anorexia nervosa (AN) is uncommon as a syndrome, despite widespread dieting or voluntary food restriction, especially among female adolescents. This suggests that restriction of caloric intake might not be the only component driving weight loss in AN. Historical observations and experimental evidence from energy expenditure studies and recordings from movement sensors reviewed in this paper reveal that AN is associated with motor activity levels and with an energy output not significantly different from that in normal-weight healthy age-matched controls. By contrast, other conditions of prolonged caloric under-nutrition are typically associated with loss of energy, slowing of movements and a decrease in self-initiated activity and well-being. Several hypotheses can be inferred from the findings: (a) that long term severe caloric restriction fails in downregulating movements and energy expenditure in AN. (b) Clinically and subjectively observable as mental and physical restlessness and continued motor activity, this restless energy, differing in intensity, seems to serve as the permissive factor for and possibly to drive exercise and hyperactivity in AN. (c) Such restless energy and increased arousal, generated sometime in the course of the weight loss process, appear to enhance the person’s self-perception and wellbeing, to heighten proprioception, to intensify body awareness and to improve self-esteem. (d) Restlessness and continued motor activity may constitute a phenotype of AN. The therapeutic value of the concept of an abnormality in the energy regulatory system, likely the result of a host of genetic and epigenetic changes in AN, lies primarily in its heuristic and explanatory power and its potential for disease prevention. Restless energy as a permissive and important component for the development and in the maintenance of AN, does not fundamentally alter treatment, since prolonged food deprivation is the principal causal factor for the development of AN. Re-nutrition within a structured treatment plan, to include individual and family therapy and, if indicated, heat application, remains the most effective symptomatic treatment for AN. Corroboration of the concept of restless activation will require the patient’s cooperation and input to identify and capture more precisely the experiences, sensations, and changes that allow the emaciated patient to remain mobile and active.

## Introduction

The success of treatment approaches in medicine depends on the correct diagnosis and a sufficient understanding of the factors underlying the symptom constellation of the disease or disorder. Regarding diagnostic clarity and understanding of anorexia nervosa (AN), its history is a story about gains and losses in knowledge.

## The Diagnostic Classifications of AN

Since its first succinct description as a clinical entity by Gull (1874, 1888) and Lasègue (1873), AN existed for a full century in its own right under the assumption that the remarkable clinical uniformity and the shared symptomatology implied a distinct, if unknown, pathogenesis.

In 1968, AN became classified as a feeding disturbance in DSM II (Dsm-II, 1968). AN reemerged in 1972 as a disorder sui generis in Feighner’s Research Criteria (Feighner et al., 1972). Specifically, Feighner’s Research Criteria refer to “periods of overactivity” and “apparent enjoyment in losing weight with overt manifestations that food refusal is a pleasurable indulgence.”

Nonetheless, from 1980, DSM III (Dsm-III, 1980) on, AN became and remains classified as an eating disorder, not only in DSM, but also in the International Statistical Classification of Diseases and related Health Problems (International Classification of Diseases [ICD], 1992).

By now, it is well recognized that the designation “anorexia” or “loss of appetite,” defined by Gull (1888) and Lasègue (1873) as a “want of appetite,” is erroneous, because true loss of appetite does not occur until late in the disease, if at all. AN might be associated with reduced appetite, but thinking and dreaming about food and/or collecting recipes reflect the physiological and cognitive presence of hunger sensations. At no time have “loss of appetite” or “anorexia” been considered defining criteria for AN. In point of fact, the International Statistical Classification of Diseases and Related Health Problems 10th Revision (World Health Organization [WHO], 2016) lists “loss of appetite” and “anorexia” as exclusion criteria.

For these reasons, the classification for the classical restricting form of AN as an “eating disorder” seems problematic. The current DSM V (Dsm-V, 2013) and ICD 10 (International Classification of Diseases [ICD], 1992) criteria with their focus on behavior prudently give greater consideration to AN as a “weight phobia leading to deliberate food restriction with pathological weight loss.” Dread of weight gain which typically develops after substantial weight loss, seems to be pathognomonic for AN.

Here we will review the evidence that in persons predisposed to AN the long lasting and severe deficiency in caloric intake and the resultant pathological weight loss engender a unique biobehavioral response, observable in physical and psychological phenomena that distinguish AN from other states of severe caloric undernutrition. We suggest that two starvation induced signs specifically, restlessness and an urge for movement, point to an anomaly in the biobehavioral regulation of the adaptation to starvation.

### Starvation and AN

The earliest descriptions of AN recognized that the degree of emaciation brought about by long term intentional reduction in food intake despite access to ample food in persons in good health had no parallel among medical disorders. The condition was found to be one of simple starvation, without signs of disease.

As in simple starvation, the prolonged and progressive caloric restriction in AN leads along with body weight loss to adaptive responses in virtually every regulatory system in the organism to guarantee survival. The blood pressure drops, the pulse rate slows down, there is hypothermia, the basal metabolic rate is reduced, there might be a heart murmur. The degree of the changes depends on the severity and duration of the caloric deficit (Pirke and Ploog, 1986). To-date, no biological markers in any of the many physiological, metabolic, or hormonal parameters altered by prolonged undernutrition have been identified that can distinguish AN from other forms of semistarvation (Hebebrand et al., 2003).

### Mood and Behavior in AN Incongruent With Mood and Behavior Observed in Semistarvation

#### The Relative Well-Being in AN

Clinicians in the 19th and in the early 20th century, who were familiar with the effects of undernutrition in the population and well aware that slow starvation was typically associated with a loss of the natural feeling of well-being (Leyton, 1946), regularly commented on the mood and behavior of persons with AN which they considered incompatible with the condition of advanced starvation.

Lasègue (1873) writes “the person with anorexia hysterica insists that she has never felt better, she does not suffer and all the worries are contradicted by her well-being.” Others refer to “a peculiar euphoric mental state” (Nicolle, 1938) and “remarkable strikingly disproportionate abundance of physical energy” (Palmer and Jones, 1939) along with a lack of concern regarding the seriousness of the profound weight loss and of its risks to the patient’s life.

Psychiatric disorders, by definition, not only disrupt a person’s functioning, but also lead to emotional suffering. By contrast, in AN the person not only continues to function, to attend school and to work, but she also asserts that she is satisfied with eating as little as possible and adjusts without complaint to the physical changes, such as a low blood pressure, low heart rate, constipation, or coldness due to the low body temperature. The person’s contentment with her condition in AN strongly contradicts the observations of others, including family and friends, who deplore the lack of food and view her emaciated body and emotional withdrawal as a sign of illness.

Bruch (1962), an astute and experienced clinician, recognized the discrepancy. She thought the absence of concern about the emaciation, the lacking awareness of fatigue and weakness corresponding to the advanced stage of malnutrition and the insistence of not being tired and of wanting to do things, to be delusional in nature, the “overactivity to be a falsified awareness of the bodily state,” and wondered about a schizophreniform pathology in AN.

In this paper we are proposing an alternate interpretation, namely that persons with AN might be accurate in reporting their unusual experiences and to consider these manifestations as actual perceptions specific and fundamental to AN (Casper, 2016). The “paradoxical sense of alertness and wellbeing” (Bruch, 1962) then might reflect a semistarvation-induced state of arousal and might be one indication that psychologically and biologically the organism predisposed to AN reacts differently to prolonged caloric deficiency leading to near total starvation compared to the organism of others, not so predisposed.

#### Restlessness and the Drive for Movement in AN

Other behavioral patterns that have attracted the attention since the earliest descriptions of AN, are restlessness and a drive for movement. Both behaviors were found in all of Gull’s (1888), Lasègue’s (1873), and Janet’s (1903) cases and have been included in many clinical descriptions since then (Casper, 1986). Albutt and Rollston (1905) mention “a strange unrest, she takes long walks far beyond her strength.” Crisp and Stonehill (1976) write “the majority of anorectics are very restless – for instance they walk or bicycle or stand whenever possible” and “the low levels of basic energy expenditure in AN appear to exist alongside a constant sense they have of restlessness which is such that they are rarely able to sit down and relax.”

It is of interest that forty percent of the participants in the Minnesota Semistarvation Experiments (Keys et al., 1950) reported some restlessness in the form of ‘difficulties in sitting still,’ yet overall “voluntary movements became noticeably slower and energy output was markedly reduced, most of the men felt weak with curtailment of self-initiated activities.” Ninety seven percent said they tired easily and movements became cumbersome, with no sign of a drive for activity and years later, the men remembered a leaden exhaustion which made exercise difficult (Eckert et al., 2018). In a 10-days supervised starvation experiment, Consolazio et al. (1967) describe the men on day 10 as in very poor condition with increased weakness and apathy toward mental and physical work.

Parenthetically, restlessness and fidgeting in AN ought not to be confused with agitation in agitated depression, a disorder within the bipolar spectrum. In agitated depression the psychomotor activation is experienced as unpleasant and undesirable and is strongly related to feelings of anxiety (Akiskal et al., 2005).

In acute AN, neither restlessness nor increased movements are the basis for complaints nor are they spontaneously mentioned. A study by van Elburg et al. (2007) suggests low awareness in AN patients; they rated themselves on physical activity, defined as restless activity, significantly lower than nurse observers.

As in other forms of advanced semistarvation (Schiele and Brozek, 1948), in AN there can be narrowing in interests, blunting of emotions, avoidance of social interactions and emotional instability, with greater self-absorption and increased self-centeredness, often hyperacuity to noise, to light and to odors.

### Continued Physical Activity and Restlessness in the Presence of Semistarvation Suggest an Energy Economy Specific to AN

The most parsimonious hypothesis which might explain continued motor activity and restlessness in AN is that normally thrifty mechanisms, which reduce physical activity and defend energy stores during times of caloric privation (Redman et al., 2008), do not operate as expected in AN, despite a 20–40% reduction in the resting metabolic rate (RMR). We have proposed that this failure to adjust energy output in AN to semistarvation might indicate a disturbance in the homeostatic mechanisms regulating energy expenditure (Casper, 2016).

Indeed, if persons with AN felt as sluggish and as feeble as the men in the Minnesota experiments after 24 weeks of semistarvation and a 25% weight loss, normal non-exercise activity (NEAT) (Levine, 2007) going to school, walking, dancing, would be a hardship and the strenuous prolonged exercise practiced by a majority of patients (Schlegl et al., 2018) with AN would hardly be possible.

## Clinical and Experimental Evidence Suggesting Specific Adjustments to Caloric Deficiency

### Well-Being and Energy Level in AN

Are there data in the literature relevant to the relative well-being and near normal motor activity and energy expenditure in AN beyond behavioral observations and the patients’ reassuring reports about their physical strength?

Research in AN, unfortunately, has been stymied by the absence of age-matched undernourished and mal-nourished low-weight female control groups.

The closest comparative data set was collected in the Minnesota Semistarvation Experiments (Keys et al., 1950). Compared with the well-being in AN, two-thirds of the men felt downhearted and had concentration difficulties. “Both subjects and observers frequently remarked that the group, lively and responsive during the control period, became apathetic during starvation, …… yet, “some spells of elation occurred……these feelings of well-being and exhilaration lasted from a few hours to several days, but were inevitably followed by low periods” (Keys et al., 1950, p. 836). In AN, by contrast, the sense of well-being seems to endure and their liveliness can often be observed when AN patients are brought for an evaluation. Unlike patients with depressive disorders, AN patients tend to be in good spirits in the early morning: nearly as many acute AN patients (87.5%) as control subjects (90%) stated “I wake up rested and refreshed” (item #3 MMPI); these numbers were slightly fewer for recovered AN patients (76.4%) (Casper, 1990).

### Self-Reports of Reactions to Starvation in AN

Overall, questions about the patients’ personal experiences and mood during the weight loss phase have not been included in assessments. Since there is rarely a chance to interview patients as they develop AN, the only approach appears to be to ask patients to describe their experiences retrospectively.

Individual narrative reports have the disadvantage that they vary from person to person, while questionnaires have the disadvantage that they enforce terms which may or may not capture the person’s experiences, given our limited knowledge about the nature of these experiences.

To give an example, a patient recalled “the exhilaration I felt from not eating, I crave it to this day. It is hard to describe. I felt powerful, I felt successful, I felt in some odd way superior. I have never had great self-esteem and not eating gave me this sense of pride. It definitely became stronger, the less I weighed. Something kicked in when I lost weight that I had not felt before.” ^∗^ This patient, it must be added, once in treatment, experienced despairing depression and hopelessness.

Patients quoted by Bruch (2001) describe similar novel feeling states with increasing weight loss: “being hungry has the same effect as a drug, and you feel outside your body. You are truly beside yourself- and then you are in a different state of consciousness and you can undergo pain without reacting…” (p. 18); another patient: “it is as if you were slowly poisoned, something like being under the chronic influence of alcohol or dope” (p. 14); a third: “It is as if you create a robot and then you can’t control what it is thinking” (p. 19), a fourth: “When it becomes a pleasure to pursue, then something else happens. One feels intoxicated, literally how I think alcoholism works” (p. 73).

These reports point to altered sensations and an altered mindset generated by undernutrition and malnutrition in AN.

### Investigations Into Energy Economy in AN

Measurements of energy expenditure and daily activity levels in AN can test the hypothesis of higher than expected energy expenditure in AN. The studies have employed different methodologies:

(a) Assessments of physical activity through interviews and self-reports.
(b) Monitoring of motor activity and fidgeting through movements sensors.
(c) Studies of total energy expenditure (TEE) measured over 14 days by the doubly labeled water (DLW) method (Schoeller and van Santen, 1982).
(d) Sleep motility recordings.
(e) Drive for activity self-assessments.

### Assessments of Physical Activity Through Interviews and Self-Reports

Compulsive exercise, ≥60 min every day, was reported in 39% of AN patients at referral (Brewerton et al., 1995). Davis (Davis et al., 1997) was the first investigator to underscore the role of activity levels in AN by studying excessive exercise prior to and during the acute phase of the disorder. Data from questionnaires showed that half of restricting AN patients reported to have been more physically active during childhood than the average girl her age and 81% exercised at least 6 h per week (“excessive exercisers”) during the acute phase of the disorder. In another study, among 80% of AN patients who qualified as excessive exercisers during the acute phase of the disorder (Davis and Kaptein, 2006), slightly more (67%) were found to have obsessive-compulsive personality traits compared to moderate or non-exercisers (59%). In a retrospective case/control comparison which included parents (Davis et al., 2005), AN patients reported higher levels of physical activity during the course of their disease compared to controls. In these studies, the majority of AN patients recalled being active during the weight loss phase of the disorder. Restlessness assessed through self-reports and observer ratings in studies by Holtkamp et al. (2003, 2004) showed relationships with leptin levels, the latter reflecting the degree of undernutrition.

Notably, clinical and outcome studies in childhood and early adolescent AN also describe excessive activity levels before or during early treatment in on average two thirds of the children which declined with weight gain (Blitzer et al., 1961; Galdston, 1974; Kron et al., 1978; Fosson et al., 1987; Kreipe et al., 1989; Steinhausen and Vollrath, 1993; Casper and Jabine, 1996).

#### Monitoring of Motor Activity and Fidgeting Through Movements Sensors and Through Self-Report and Observer Ratings

Many investigators, interested in the occurrence of hyperactivity and exercise in AN, obtained measurements of daily activity levels – defined as any bodily movement produced by skeletal muscles that requires energy expenditure – through movement sensors (Actiwatch; Actigraph; Actiheart; Sensewear armband). Physical activity levels in hospitalized or ambulatory AN patients before or during early treatment using movement sensors were found to be equal or higher than in normal-weighed controls(Bouten et al., 1996; van Marken Lichtenbelt et al., 1997; Harris et al., 2008; Hechler et al., 2008; Bratland-Sanda et al., 2010; Hofmann et al., 2014; Keyes et al., 2015). Comparison ratings using self and observer assessments (Davis et al., 1994; Bossu et al., 2007; Ehrlich et al., 2009; Long et al., 2009) found no differences in reported activity levels between patients with AN and control subjects. Importantly, van Elburg et al. (2007) on finding significant correlations between nurses observations of activity levels and patients’ actometer readings, showed that patients significantly underestimated their physical activity when compared to nurses’ ratings, suggesting that patients might be less reliable judges of their own movements.

In a study by Gianini et al. (2016), however, which used a novel “intelligent device for energy expenditure and activity,” AN patients were less active at low weight than after weight gain or healthy controls; the weight gain group in this study showed considerable attrition. Yet, fidgeting, assessed as changes in body position while seated, showed no difference between patients at low weight, weight restored or healthy controls. The same group (Belak et al., 2017) measured fidgeting in hospitalized AN patients using a shoe-based monitor and found the patients’ fidgeting behavior to be 1.7 times greater than that of healthy controls.

#### Total Energy Expenditure (TEE)

Four studies of patients with acute AN diagnosed by DSM IV criteria which measured TEE using the doubly labeled water method (DLW) found, despite significantly lowered RMR, no differences in total energy expenditure between AN patients and healthy normal weighed age-matched controls (Casper et al., 1991; Pirke et al., 1991; Bossu et al., 2007; Zipfel et al., 2013); two studies conducted in chronic AN observed slightly lower than normal TEE (Platte et al., 1994; van Marken Lichtenbelt et al., 1997).

#### Sleep Motility Recordings

Crisp et al. (1971) measured movements during sleep in AN patients before and after treatment. Nocturnal motility recordings in acute AN were high during sleep and approximately halved after weight gain. The greatest restlessness was observed during the first part of the night. They comment that “the nocturnal restlessness of these patients is an aspect of their total restlessness which may be biologically determined.”

#### Drive for Activity Self-Assessments

Sternheim et al. (2015) measured “drive for activity” in AN patients and observed a relationship to measures of eating pathology. In another study (Keyes et al., 2015), AN patients reported a higher drive to exercise than controls, despite similar actometer-recorded physical activity levels.

To summarize, these reports reveal little if any reduction in total daily energy expenditure or in daily motor activity in AN, despite profound weight loss. In view of the lowered RMR, proportionately more energy is expended in movements in AN than in normal controls.

## “Wellbeing and Restless Energy” as Permissive Factors for Body Image and Body Perception Changes Enabling the Personal Focus on the Body

Anorexia nervosa stands out as a disorder in which the principal symptom, a strong commitment to restriction of food intake, corresponds to the person’s wishes. In most contemporary cases the goal of eating less is to achieve slenderness. Nevertheless, the literature shows that motives can differ. For religious young women abstinence from food was part of their asceticism to enhance spirituality and to renounce pleasures (Bell, 1985). For a few, depressive feelings or uneasy stomach sensations diminishing the appetite may start the process (Lee et al., 1993). A prepubertal boy ate less and less, because he discovered that he felt lighter and more agile on the tennis court. Indeed, Touyz et al. (1993), have shown that in men and women AN can be triggered by a pursuit of exercise.

The typical time of onset for AN is adolescence, emotionally a time of separation, of reaching out and developing new bonds and relationships beyond the family.

Developmentally, adolescence with its growing psychological sensitivity and cognitive abilities constitutes a time of gradual consolidation of the person’s self-concept. Self confidence and trust, the ability to depend on people and to feel secure through early caring interactions in the family are the building blocks for friendships and relationships beyond the family. The healthy adolescent seeks independence from parental control, yet at the same time seeks to maintain the security of the parent’s love and support. In friendships and peer relationships the adolescent seeks to anchor herself within her female group of friends while testing relationships with boys.

In psychological terms then, in most cases, AN can be conceptualized as a failed separation/individuation attempt, where, differing in degree, early traumatic experiences have undermined the person’s confidence to rely emotionally on parents and friends. Sometimes, if fears of puberty lead adolescents to avoid eating meals to arrest growth, the psychological issues seem to be less pervasive and easier to overcome.

Instead of unfolding within personal relationships, the struggle for independence and self-sufficiency in AN seeks independence and self-sufficiency through renouncing food and in the process creates a new self in unity with the body. Emotional conflicts find expression in the patient’s relationship to her body which seems to take over the function of affect regulation and for a sense of self (Casper, 1987a,b). Dieting treats the body like an object in need of limits and controls, a repetition perhaps of how the person has felt in relationships: not accepted unconditionally along with her shortcomings, but in need of change to be accepted. The personal sense of self appears to become to a large extent identified with the slim, and ultimately, wasted body in AN. Developmental and personal factors converge to consolidate this newly created self-body unit.

One intriguing issue is the nature of the person’s involvement with her body as a source of gratification. Through adopting her body as an object of constant attention and satisfaction, the person becomes more and more involved and identified with her changing body, expressed by one patient in this way: “my body is not an idea of mine, but my idea of my body is an idea of mine.”

Persons with AN develop an irrational attachment to their thin body with some going as far as to admire their skeletal shape in privacy. This symptom of “mirror gazing” reveals how much the person with AN cherishes her wasted body and assumes that others share this approval when they display their skeletal body by dressing sparingly. “Mirror gazing” indicates that AN patients can see their bony frame visually, realize what is cognitively acceptable, but might not register and connect the visual information into a bodily feeling and process its implications into full awareness.

Restless movements and continued physical strength despite severe weight loss and the ensuing sense of feeling in command are likely to intensify proprioception and contribute to keener body perception. Feeling restless energy appears to counteract the unpleasantness and discomfort of the many physical changes associated with semistarvation re-enforcing the assertion that all is well. The sustained energy could also relegate awareness of the original disappointments and conflicts into the background.

Importantly, the restless energy in AN might be essential for the incomplete adjustment of body size perception. Studies have shown a positive relationship between body size overestimation and weight loss, loss of appetite and denial (Crisp and Kalucy, 1974; Casper et al., 1979). At most, AN patients overestimated their wasted size back to their size at normal weight. This illusionary larger size of their bony frame, a function of starvation severity, seems to dissimulate the realization that the severe weight loss puts the patient’s life at risk. The imagined larger size and changing sensations on weight gain might elucidate why any weight increase from the low weight ominously triggers morbid fears of weight gain and feelings of fatness, the latter symptoms more specific to AN in Western culture as opposed to the more body focused fear of unpleasant sensations of gastric bloating and distress in Eastern culture (Lee et al., 1993). This interpretation would be supported by observations (Crisp and Kalucy, 1974; Casper et al., 1979) that a more accurate appraisal of the body image is associated with a better prognosis and a less severe illness.

Conversely, the physiological and hormonal adaptations to the profound changes in body weight and nutrition remain tightly linked to the degree of starvation.

As such, normal or higher than normal energy expenditure in AN points to a rare biologically based imbalance in the energy regulatory system. If so, this might shed light on the observation, that despite widespread dieting among adolescent girls in Western Society only about one percent develop AN.

## Treatment Implications

Unlike the treatment of other psychiatric disorders, where symptom removal provides relief from suffering, the situation in AN is entirely different. With the principal sign ostensibly being pathological weight loss, the recommendation to eat more caloric food to reverse the weight loss, increases anxiety and suffering in the patient. Not surprisingly, treatment meets with resistance. Unlike in famine, where the person wants food, the patient with AN maintains that she is comfortable with her emaciated body, there is no desire to eat more to regain weight, even if treatment leads to better physical health.

Still, given that food deprivation generated the disease, food remains the best and most effective treatment for AN. There is wide agreement that without weight restoration to within the normal weight for age and height, full recovery from AN is not possible. Providing energy in the form of food or heat would be expected to reduce restlessness, but, less desirably, along with it also reduce the sense of liveliness and restless energy.

Consequently, the assumption of a facilitating role of the energy imbalance for the development of AN does not fundamentally alter the treatment plan. The therapeutic value of the concept of an abnormality in the energy regulatory system, likely the result of a host of genetic and epigenetic changes in AN, lies primarily in its heuristic and explanatory power and its potential for disease prevention.

As mentioned before, restless energy may play some role in facilitating the weight loss in AN. If indeed the restless energy level contributed to the patient’s acceptance of her wasted body and her defense of her low body weight, then exploring the reduction of these sensations with weight gain and offering tentative explanations, might be a starting point for a conversation. The aim is to engage the patient on a personal level and to encourage self-observation and self-inquiry.

Furthermore, if we accept the proposition that restless energy and activity level and the person’s overall wellbeing depended in large part on the negative energy balance, then with increased food intake and a positive energy balance, the decline of energizing sensations would unmask the unhappiness and emotional conflicts which were at the root of the initial food restriction. The recognition that the patient might be feeling less well physically and feel psychologically defeated, having yielded to pressure to eat more, can help us understand that the patient might be at the highest risk for despair and might need as much emotional support and physical comfort as possible to help her tolerate the weight gain.

Another set of symptoms, seemingly distortions, the often reported complaint of feeling fat, of feeling heaviness and sluggishness after eating caloric food or after experiencing small amounts of weight gain, might well be related to the diminishing energizing sensations and the reduced drive for activity. By understanding their biological nature, these sensations might be experienced as less ominous. Offering an explanation for such alarming sensations on gaining weight, might be a starting point for discussing personal fears and conflicts. In view of the importance of movement to patients emotionally and physically as they gain weight, the treatment plan ought to include opportunities for regulated activity. Learning of a biobehavioral defect might decrease the parents’ helplessness in reaching out to their child, knowing that forces beyond mere willpower might have been at work to support their child’s stubborn opposition to eating.

Providing energy in the form of heat to the patient as recommended by Gull to improve food uptake (Gull, 1874) would be expected to decrease restlessness and overactivity and provide physical comfort. A study by Bergh et al. (2002) that prescribed rest and warmth, regulated by the patient to up to 40°C for 1 h after each meal in a structured treatment plan reported high remission rates after 1 year. Their design restricted physical activity, leaving the question about possible changes in activity open. Another study reported antianxiety effects of warming in AN (Zandian et al., 2017). Gutierrez et al. (2008), who have postulated a disordered thermoregulatory response in AN, reported significant clinical improvement in three AN patients, who were prescribed heat in different forms (Gutierrez and Vazquez, 2001). By contrast, an earlier study found no differences in weight gain in AN patients, who were wearing warming vests (Birmingham et al., 2004). The beneficial and therapeutic factors in warming the patient appear to be the reduced energy needs, the physical comfort and calming influence and importantly, the patients’ assent and control of the heat treatment.

Previous work has shown that the insidiousness and tenacity of AN depends on the person’s emotional health, the cohesion and emotional health of the family and the inherited disposition to psychiatric disorders. The psychological problems differ from person to person and family to family. The co-morbid psychiatric diagnoses can range from an adjustment reaction to a combination of psychiatric disorders, though rarely bipolar disorder or schizophrenia. In other words, if fear of sexual maturation in the presence of a fairly good self-esteem, in a supportive family triggers dieting leading to AN in a teenager without a predisposition to psychiatric disorders, then the chances for full recovery are good. In these instances, AN psychologically reflects a temporary emotional maladjustment and not a serious deviation from normal development. Tolerating weight gain is made easier if parents can understand their child’s dilemma and accept and support their child’s needs.

Lastly, regarding disease prevention, the hypothesis of restless energy in AN suggests that high activity levels in childhood might be risk factors for AN in the dieting teenager, particularly, if continued food restriction does not lead to a decline in the urge for movement.

## Conclusion

This is a critical analysis of neglected symptoms fundamental to AN. Clinical reports and the reviewed studies corroborate restlessness and continued motor activity as integral symptoms of AN. The lack of significant lowering of energy expenditure during the acute phase of AN supports the theory that the mechanisms regulating energy economy in AN may be different from those in other starvation states during experimentally induced caloric restriction or famine, where energy output is progressively lowered to protect the body from excessive weight loss. Given the reduced RMR, proportionately more energy is expended as activity in acute AN, manifest as “agreeable restlessness” (Scheurink et al., 2010) and spontaneous activity.

As a unifying theory, this hypothesis of continuous restless energy contributing to activity levels and well-being in AN helps explain many of the symptoms of AN and with further research can contribute to identifying a homogenous group of AN patients, who would be expected to be heterogenous with regard to co-morbid psychopathology. If persons predisposed to AN share a common abnormality triggered by the regulatory adaptations to the caloric deficiency, then this defect would remain dormant at a normal weight and would only become manifest under conditions of food deprivation creating a prolonged negative metabolic balance. In regard to an underlying genetic structure, the hypothesis allows for the possibility that genes contributing to foraging, to food anticipatory activity and in migratory restlessness in animals as well as genes involved in hypothermia may contribute to spontaneous activity in AN.

The proposals put forward by Epling and Pierce (1988) which differ from ours, raise interesting questions. For example, the assumption that the activity-based anorexia induced in the experiment of Routtenberg and Kuznesof (1967) simulates “self-starvation” in AN does not take into account that the severe food restriction imposed on rodents leading to activity-based anorexia has not been observed under natural conditions. As Mrosovsky (1984) commented some time ago, since the activity-based anorexia model in rodents is the result of a laboratory experiment, it does not reflect “self-starvation.” Despite these limitations, the activity-based anorexia rodent model remains the most informative animal analog of AN, albeit excessive wheel running, not food restriction, ultimately leads to death in the underfed animals.

In another premise, Epling and Pierce (1988) describe “persons with AN as a naturally selected group, who became active during times of food restriction.” Taking an evolutionary perspective, this theory would ensure survival, but does not explain for how long such a cohort remains active without access to food nor why males would be excluded from natural selection. As Levine (2015) succinctly states “all species that have been studied show similar responses to starvation: first, there is a short-term increase in non-exercise activity thermogenesis or NEAT (ascribed to foraging), but once starvation is prolonged, NEAT decreases—even in fish.”

Finally, if an abnormal adaptation of the energy regulatory system to severe undernutrition underlies AN, how can this phenomenon be further investigated?

Unfortunately, ethical and practical considerations preclude experiments which could test the effects of severe and prolonged undernutrition on physical activity levels in healthy female adolescents. Many researchers have been aware of higher than expected activity levels, yet standard instruments assessing the patients have not included questions regarding an urge for movement or restlessness. A first step, therefore, might be to determine whether AN patients are aware of and to what extent they experience “an increased urge for movement” or “physical and of mental restlessness” during the weight loss phase. A questionnaire could be developed to obtain information retrospectively about the nature of the patients’ sensations and experiences during the weight loss phase, their activity and energy levels, and their participation in daily functions and activities. In a second phase, responses to the questionnaire at the time of the diagnostic evaluation before treatment is started, could be linked to actual measurements of spontaneous activity through motion sensors and studies of daily energy expenditure.

## Ethics Statement

The quote on p. 7 was obtained as part of the Follow-up Study (Casper and Jabine, 1996). The study was approved by the Human Subjects Committee of the University of Chicago. Written informed consent was obtained from each patient after the study had been fully explained to them.

## Author Contributions

The author confirms being the sole contributor of this work and has approved it for publication.

## Conflict of Interest Statement

The author declares that the research was conducted in the absence of any commercial or financial relationships that could be construed as a potential conflict of interest.
